# Mathematical modeling identifies potential gene structure determinants of co-transcriptional control of alternative pre-mRNA splicing

**DOI:** 10.1093/nar/gky870

**Published:** 2018-10-01

**Authors:** Jeremy Davis-Turak, Tracy L Johnson, Alexander Hoffmann

**Affiliations:** 1 San Diego Center for Systems Biology (SDCSB), University of California, San Diego, La Jolla, CA 92093, USA; 2 Department of Molecular, Cell, and Developmental Biology, University of California, Los Angeles, CA 90095, USA; 3 Molecular Biology Institute (MBI), University of California, Los Angeles, Los Angeles, CA 90095, USA; 4 Department of Microbiology, Immunology, and Molecular Genetics (MIMG), University of California, Los Angeles, CA 90095, USA; 5 Institute for Quantitative and Computational Biosciences (QCB) University of California, Los Angeles, Los Angeles, CA 90095, USA

## Abstract

The spliceosome catalyzes the removal of introns from pre-messenger RNA (mRNA) and subsequent pairing of exons with remarkable fidelity. Some exons are known to be skipped or included in the mature mRNA in a cell type- or context-dependent manner (cassette exons), thereby contributing to the diversification of the human proteome. Interestingly, splicing is initiated (and sometimes completed) co-transcriptionally. Here, we develop a kinetic mathematical modeling framework to investigate alternative co-transcriptional splicing (CTS) and, specifically, the control of cassette exons’ inclusion. We show that when splicing is co-transcriptional, default splice patterns of exon inclusion are more likely than when splicing is post-transcriptional, and that certain exons are more likely to be regulatable (i.e. cassette exons) than others, based on the exon–intron structure context. For such regulatable exons, transcriptional elongation rates may affect splicing outcomes. Within the CTS paradigm, we examine previously described hypotheses of co-operativity between splice sites of short introns (i.e. ‘intron definition’) or across short exons (i.e. ‘exon definition’), and find that models encoding these faithfully recapitulate observations in the fly and human genomes, respectively.

## INTRODUCTION

Eukaryotic genes are organized into coding exons, separated by non-coding intron sequences that are removed from the pre-messenger RNA (mRNA) via splicing to produce the mature mRNA. Distinct mRNA isoforms may be generated from a single gene by alternative splicing (AS). AS is a critical mechanism that vastly expands transcriptome and proteome diversity and regulates many biological functions, including development and differentiation. Misregulation of AS is implicated in many diseases including neurological disorders and cancer (1,2). One of the most commonly studied forms of AS is exon skipping in which a single so-called ‘cassette exon’ is either included or excluded in the mature mRNA.

AS may generate cell-specific, tissue-specific or context-dependent mRNA isoforms. Substantial advances have been made in identifying the *cis*-elements and trans-acting factors that regulate AS in a variety of physiological contexts and in several organisms. In mammalian cells, splicing enhancer and repressor sequences have been identified, as well as cognate RNA-binding proteins (RBPs) that may function to enhance exon inclusion or skipping (3). With the advent of genome-wide mRNA measurements, efforts are under way to identify the binding sequences and locations of all human splicing factors, and characterize their functions in determining mRNA isoforms, e.g. (4). The resulting genome-wide data can form the basis for computational modeling of the so-called splicing code to predict mRNA isoforms in a variety of cell types or conditions. To date, the most successful models have employed a statistical learning approach that side-steps an understanding of underlying molecular mechanisms (5,6). Interestingly, these models involve not only RBP–RNA interaction parameters but also a variety of genetic and epigenetic features as input parameters for the statistical learning algorithm. The importance of such parameters in the model suggest that gene structure, chromatin and RNA polymerase play a role in determining splicing outcomes. However, the mechanistic underpinnings of these statistical parameters remain unclear although emerging research has provided qualitative links between AS and chromatin-associated events (7,8).

Recent studies have revealed splicing as a largely co-transcriptional process, which is usually initiated and sometimes completed on the chromatin and during the RNA polymerase elongation phase (9–12). This realization has led to the formulation of dynamical co-transcriptional splicing (CTS) models (13–17), which allow exploration of how intron excision efficiency is a function of gene structure, polymerase dynamics and other chromatin events during CTS. However, these models cannot (yet) be used to study AS. Alternative splice site choice may be affected when splicing is co-transcriptional, as the Pol II elongation rate determines the timing of splice signal availability and competition between splice sites (18–22). Further kinetic complexities may arise when there is cooperativity between neighboring splice sites across introns or exons, as proposed as part of the intron or exon definition hypotheses (23).

In this study, our aim was to develop a foundation for mathematical modeling co-transcriptional alternative splicing (CTAS), in order to relate gene structure, polymerase dynamics and splice site co-operativity to the propensity for an alternatively spliced cassette exon. We provide two formulations of the CTAS model. The more complete formulation encodes distinct events at the 5′ and 3′ ends of each intron and a joining event. This formulation can easily be executed to test AS scenarios requiring six or fewer splice sites, but becomes intractable when large number of introns must be considered. The CTASsimple formulation assumes that the reactions at the 5′ and 3′ ends are fast, such that they do not need to be kinetically described, resulting in a simplified computation. This formulation enables us to simulate splicing of gene isoforms containing up to nine introns, encompassing the splicing landscape of over half of all human genes. We show that the model can recapitulate important regulatory consequences of both exon definition and intron definition (24,25), and can additionally be used to simulate the effects of RNA polymerase-mediated recruitment of splicing factors (26,27).

## MATERIALS AND METHODS

### Model description

The model is represented as a Markov Chain (MC) with transition matrix Q and is always a Directed Acyclic Graph. The root node represents the premature transcript with all introns present and all other nodes represent unique states of splicing outcomes. The absorbing states in the MC are all fully spliced products. The edges connecting the nodes represent splicing-related reactions, either splice site availability or pairing reactions. Availability reactions at 5′ and 3′ splice sites of introns a and b proceed with rates *k*_5′(a)_ and *k*_3′(b)_, respectively. Pairing reactions between a 5′ splice site of exon (a) and 3′ splice site of exon (b) (subject to b ≥ a) can proceed (at rate *k*_pa–b_) only if the availability reactions have occurred at the respective splice sites.

For a gene with N introns, there are N–1 internal exons, and thus 2^N–1^ possible fully spliced products, because each internal exon can either be included or excluded. Each of the 2^N–1^ fully spliced isoforms is represented by a subset S_f_ of absorbing nodes. All such subsets S*_k_*∀ *k* ∈ [1, 2, …, 2^N − 1^] are non-overlapping. This interpretation is necessary because for each of *n* exons that was skipped in a given isoform, there are two splice sites adjacent to that exon that were not used in any splicing reaction, yet an availability event may have occurred at each of those splice sites. Thus, the subset S*_k_* for isoform *k* that skips *n* exons contains 4*n* nodes.

Each node *x* is represented by a lexographically ordered series of reactions that it has undergone. Let R be an upper diagonal matrix entries of all the pairing reactions. R*_ij_* represents a pairing of the 5′ splice site from intron *i* to the 3′ splice site of intron *j*: *i* ≤ *j*. A constitutive splicing reaction occurs when *i* = *j*. There are *N*(*N* + 1)/2 splicing reactions possible. We denote the identity of each node by a tuple of availability reactions states and entries into R. For example, a splicing intermediate in which intron 1 is constitutively spliced and the donor of intron 2 has spliced to the acceptor of intron 3, and the availability reactions have not occurred at the unused splice sites, is denoted:

\begin{equation*}{{x}}_{{{{r}}^{{5}}_{{1}}}{{,}}{{{r}}^{\rm{3}}_{\rm{1}}}{\rm{,}}{{{r}}^{\rm{5}}_{\rm{2}}}{\rm{,}}{{{r}}^{{\rm{ - 3}}}_{\rm{2}}}{\rm{,}}{{{r}}^{{\rm{ - 5}}}_{\rm{3}}}{\rm{,}}{{{r}}^{\rm{3}}_{\rm{3}}}{\rm{,}}\left( {{\rm{1,1}}} \right)\left( {{\rm{2,3}}} \right)}.\end{equation*}

where $r_j^5$ and $r_j^3$ indicate recruitment at the 5′ and 3′ splice sites, respectively, of intron *j* have occurred, and $r_j^{-5}$ and $r_j^{-3}$ indicate that they have not.

### Simulations of transcripts of model genes

To simulate each phase of the splicing of a transcript, we require a vector *β* of starting probabilities, such that

\begin{equation*}\overrightarrow {{P_\beta }} \ \left( t \right) = \ \beta {e^{ - Qt}}\end{equation*}

with *β*_a_ is the probability of starting in state a, and $\overrightarrow {{P_\beta }} ( t )$ is the probability of being in state b after time *t* given $\beta$ and *Q*, the transition matrix.

Simulating post-transcriptional splicing (PTS) is equivalent to letting the simulation run until *t* →∞. In this case, the solution can be found by setting all diagonals of *M* that correspond to the non-zero eigenvalues of *Q*, to 0. To simulate purely PTS, meaning that no CTS is allowed, we use the full *Q* of *N* introns and the initial *β*_0_:

\begin{equation*}\ {\beta _{ij}} = \ \left\{ {\begin{array}{@{}*{1}{c}@{}} {1\ {\rm{if\ }}i\ = \ 1}\\ {0{\rm{\ otherwise}}} \end{array}} \right.\end{equation*}

To simulate CTS, the simulation is broken up into phases corresponding to the time intervals during which certain subsets of splicing reactions are available. The elongation rate and the distance between splicing elements determine the duration of each phase. For example, after the first intron is transcribed, only intron 1 can be spliced while RNAPol processes through the downstream region, until intron 2 is transcribed. We shall denote this duration as *T*_1_.

Starting first with *N* = 1, we use the initial ${\beta _0} = \ [ {10} ]$ and compute:

\begin{equation*}\ {\theta _1} = \overrightarrow {{P_{{\beta _0}}}} \ \ \left( {{T_1}} \right) = \ {\beta _0}{e^{ - {Q_1}{T_1}}},\end{equation*}

which simplifies to:

\begin{equation*}\left[ {\begin{array}{@{}*{1}{c}@{}} {{e^{ - {k_1}{T_1}}}}\\ {1 - {e^{ - {k_1}{T_1}}}} \end{array}} \right],\end{equation*}

where *k*_1_ is the rate of constitutively splicing the first intron.

Next the vector *β*_1_ is constructed from the results of simulating phase I:

\begin{equation*}\ {\beta _{1\ }} = \ \left[ {{\theta _2},\ {{\vec{0}}_{y2}}} \right],\end{equation*}

where ${\vec{0}_{y2}}$ is a vector of length *y*_2_ = rows(*Q*_2_) − rows(*Q*_1_). This vector is then used to simulate phase II:

\begin{equation*}\ {\theta _2} = \overrightarrow {{P_{{\beta _1}}}} \ \ \left( {{T_2}} \right) = \ {\beta _1}{e^{ - {Q_2}{T_2}}}.\end{equation*}

For a gene with *N* introns, this strategy is then iteratively followed until we have computed $\theta$*_N_*, which is the vector of probabilities of each state after CTS. Note that *T_N_* corresponds to the time it takes to elongate the final exon. Once this CTS vector has been calculated, we simulate PTS as detailed above, starting with *β* = *θ_N_*, to obtain $\theta$^complete^, which gives us the probability of obtaining each of the 2*^N^*^− 1^ fully spliced isoforms. ($\theta$^complete^ will be 0 for all transient states of the matrix, which represent the intermediates containing one or more introns. We obtain the following expression for $\theta$^complete:^

\begin{eqnarray*}\ {\theta ^{\rm complete}} &=& \left[ {\left[ {\left[ {\left[ {{\beta _0}*{e^{ - {Q_1}{T_1}}},{{\vec{0}}_{y2}}} \right]*{e^{ - {Q_2}{T_2}}},{{\vec{0}}_{y3}}} \right] \ldots \ {{\vec{0}}_{yN}}} \right]*{e^{ - {Q_N}{T_N}}}} \right]\nonumber \\ && \, *{e^{ - {Q_N}\infty }}\end{eqnarray*}

### Simulations of human and fly transcriptomes

For both *Homo sapiens* and *Drosophila melanogaster*, we selected 100 RefSeq genes at random from each group of genes containing 2–6 exons (500 genes total per species). Care was taken to ensure that the lengths of exons and introns were indeed representative of all genes in each class. The lengths of all introns and exons were used as input for the model to calculate ${\theta ^{\rm complete}}$: the time vector $\vec{T}$ was determined length of each gene element in combination with the default elongation rate *E*, determined, where *T*_1_ = *E* * (length of exon 2 + intron), *T*_2_ = *E* * (length exon 3 + intron 3), etc.

### Modeling the splicing joining rate in the case of intron definition (IS)

DNA looping studies have demonstrated a log-linear relationship between looping distance and free energy associated with looping (28). Because calculating kinetic rates involves taking the exponential of a free energy term, kinetic rates of DNA looping therefore are linearly related to distance. Similarly, intron definition generally penalizes introns over 200 nt in length (29). To adapt this concept to looping of pre-mRNA undergoing splicing, we also take into account the propensity of RNA to take on secondary structures via base-pairing interactions by using the square root of distance as the penalty. This formulation results in the following function for *k*_p_ of an intron as a function of its length *L*, the default splicing rate *k*_p0_, and the 200-bp minimum:

\begin{equation*}kp\ \left( L \right) = \ kp0*\ {\left( {\frac{{200}}{{{\rm{max}}\left( {L,200} \right)\ }}} \right)^{1/2}}.\end{equation*}

The model is available for download at https://github.com/jdavisturak/AltSplicingModel.

## RESULTS

### A mathematical model reveals that co- but not post-transcriptional splicing favors a default splice pattern

**Figure 1.**
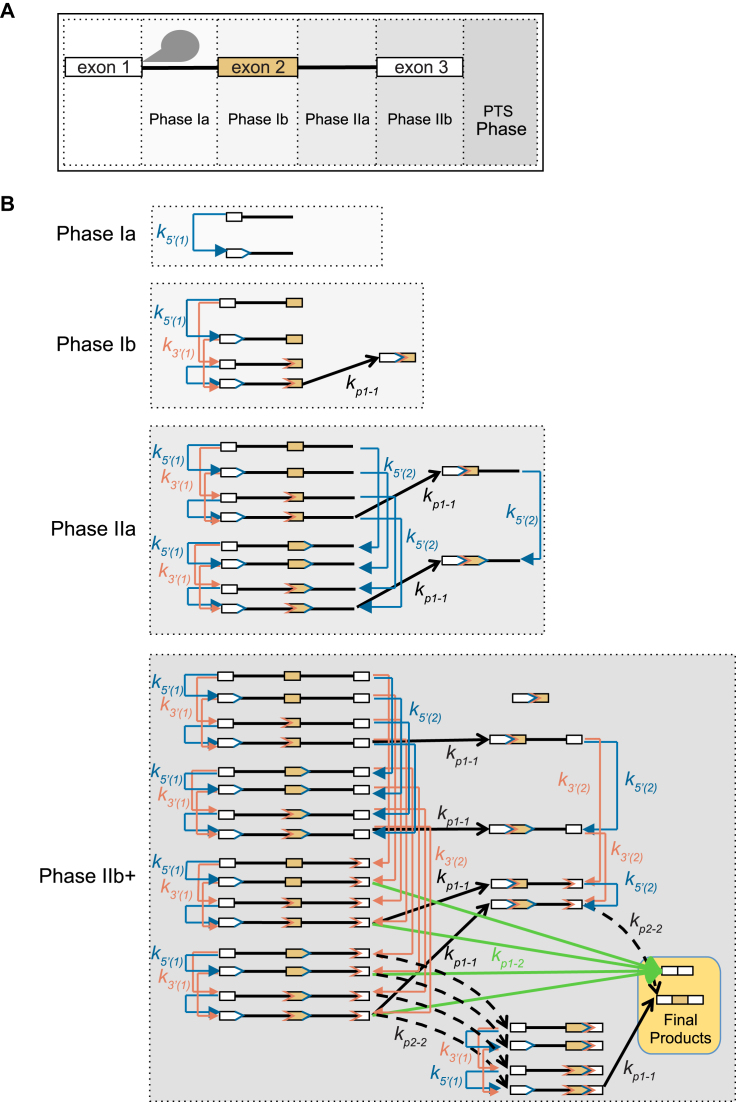
A model of CTS. (**A**) Diagram of transcriptional phases used to simulate the two-intron model used for testing cassette exons. (**B**) Splicing-related reactions that occur in each transcriptional phase. Blue and orange lines indicate 5′ and 3′ availability reactions (*k*_5′(i)_ and *k*_3′(i)_), respectively, and 5′ or 3′ splice sites that have become available are indicated by blue and orange chevrons, respectively. Availability reactions describe the deposition of small nuclear RNAs and interacting proteins with each splice site, thus rendering it available to engage in pairing and excising reaction. Black arrows indicate constitutive pairing reactions: solid black arrows correspond to intron 1 removal (*k_p_*_1–1_) and dashed black arrows correspond to intron 2 removal (*k_p_*_2–2_). Green arrows correspond to the alternative pairing reaction (*k_p_*_1–2_). Reaction rates can either be varied or kept constant across all phases in modeling simulations.

To develop a model of co-transcriptional of alternative splicing (CTAS), we first mapped out the reaction diagram (Figure 1). In principle any 5′ splice site can join with any downstream 3′ splice site. However, splice sites first must be synthesized by an elongating polymerase (defined by the phases in Figure 1), and the RNA must undergo rearrangements to become ‘available’ for participating in a pairing and excision reaction. The ‘availability reaction’ accounts for the need to recruit small nuclear RNAs and interacting proteins, or to undergo spliceosome-mediated rearrangements, and is related to splice site commitment. However, the availability reaction does not necessarily commit the available splice site to a specific pairing and excision event with a specific partner splice site. The model formulation is therefore able to account for prior observations where splice site commitments on a given gene may occur in a different temporal order than intron excision (30). Thus, splicing of introns requires three steps: 5′ splice site availability, 3′ splice site availability and pairing of available splice sites, and each state in the MC corresponds to a unique intermediate step. The model assumes that elongation during a given phase is independent of transcriptional initiation and has a fixed duration. The probability of changing states in a given time period is therefore a function of the rates of all possible reactions from that state, and can be simulated with a continuous-time MC (see ‘Materials and Methods’ section).

We first simulate phase Ia, in which the only possible kinetic reaction incorporates the steps leading to the availability of the 5′ splice site of intron 1. The resulting probabilities are then used as starting conditions for phase Ib. This strategy is repeated until the final phase, where we simulate the model for a duration approaching infinity. As a result, the probability densities reside only with the absorbing states of the model (which correspond to transcripts with no intron). Thus, for the 2-intron case demonstrated in Figure 1, the output of the model is the fraction of isoforms that have 0 or 1 intron removed. For the 3-intron case, for example, the output is the fraction of isoforms that have 0, 2, or the case of either the first or second intron removed (see ‘Materials and Methods’ section).

As the combinatorial expansion of splice sites renders this model intractable when considering more than six splice sites, we also constructed a modified version of the model (CTASsimple) where the 5′ and 3′ splice sites availability reactions are not explicitly considered rendering the catalytic splicing reaction, the sole regulatory reaction. This contraction greatly simplifies the computations and allows us to simulate genes containing up to nine introns.

**Figure 2.**
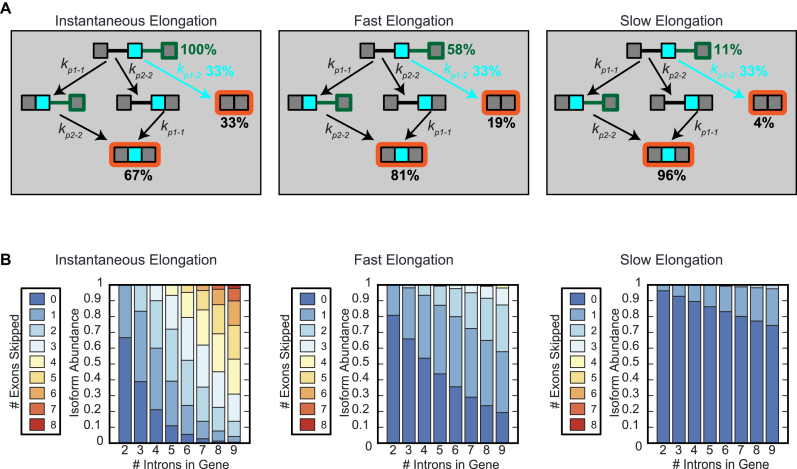
CTS increases exon inclusion fidelity. (**A**) Diagram of splicing outcomes when elongation is instantaneous (left), fast (middle) or slow (right). Cassette exon inclusion was calculated using the simplified model, with all pairing reactions (*k_p_*_1–1_, *k_p_*_1–2_ and *k_p_*_2–2_) occurring with equal rates. Green lines/outlines indicate newly formed transcript with the green percentages indicating the proportion of transcripts that still contain intron 1 when the green lined region is finished being transcribed (phase I). During phase II, the three rates compete equally, thus 33% of the unspliced transcripts skip the cassette exon (blue percentages). Percentages in black next to orange boxes indicate proportion of the transcripts that end up in either fully spliced isoform. (**B**) Calculated distributions of isoforms of model genes expanded from 2 to 9 introns. Isoforms were grouped by number of exons skipped. All phase times were either instantaneous (left), fast, 0.55 min (middle) or slow, 2.2 min (right).

To examine the quantitative aspects of the CTASsimple model, we first simulated splicing patterns in model genes under various elongation conditions. For a simple gene with one potentially alternative exon, the alternative reaction has to compete with two constitutive reactions. If CTS does not occur and all pairing rates are equal, then the middle exon will be skipped in 33% of the transcripts (Figure 2A, left). However, if CTS (i.e. non-instantaneous elongation in the model) occurs, there will be a small window during which the 5′ splice site of the first intron may be paired to the 3′ splice site of the first intron, but not to the second intron. When elongation is relatively fast or slow (e.g. 6 kb/min or 1.5 kb/min), exon skipping ranges from 19 to 4% in the model gene (Figure 2A, middle and right, respectively). This general principle also applies to genes with more than two introns where perfect fidelity (all exons included) falls off as a function of intron count, and decreasing the elongation rate dramatically decreases the fraction of transcripts that skip many exons (Figure 2B).

These results indicate that when transcripts are primarily spliced co-transcriptionally, the default mode of splicing includes all exons. In contrast, if the CTS rate is low and therefore most transcripts are spliced post-transcriptionally, then the propensity for exon skipping increases, and no predominant default splice pattern in the mature transcript emerges.

### Gene structure determines the default splice pattern and the regulatability of cassette exons

**Figure 3.**
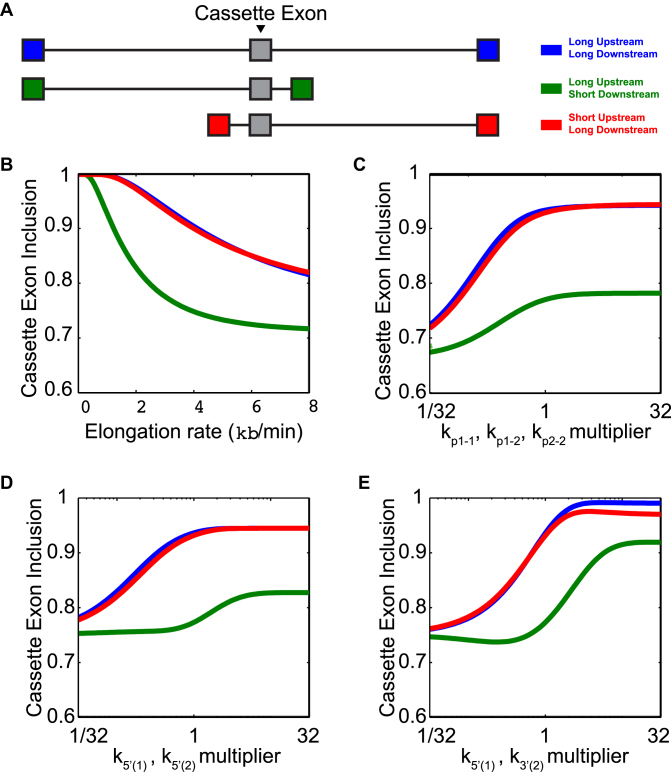
The interplay between gene structure and kinetic parameters. (**A**) Schematic diagram of three example genes representing various splicing scenarios. Gray exons are the cassette exons and colors correspond to the three scenarios of intron sizes to be spliced, long upstream and downstream introns (blue), long upstream and short downstream introns (green), and short upstream and long downstream introns (red). (**B**–**E**) Cassette exon inclusion in the three genes is plotted as a function of elongation rate (B), rate of pairing available splice sites (*k_p_*_1–1_, *k_p_*_1–2_ and *k_p_*_2–2_) (C), rate of 5′ availability (*k*_5′(1)_, *k*_5′(2)_) (D) or rate of 3′ availability (*k*_3′(1)_, *k*_3′(2)_) (E).

We next investigated the relationship between gene structures and exon inclusion using the full CTAS model. A typical long intron (3 kb) downstream from a cassette exon resulted in high exon inclusion (94 or 82% at 3 or 8 kb/min, Figure 3A and B, blue and red), whereas a short downstream intron resulted in a decreased inclusion rate regardless of the speed of elongation (78 or 72% at 3 or 8 kb/min, Figure 3A and B, green). In contrast, the length of the upstream intron (blue versus red) had little effect on inclusion.

In addition, as the rates of splice site pairing (*k*_pair_), 5′ availability (*k*_5prime_) or 3′ availability (*k*_3prime_) increase, cassette exon inclusion increases as well, and a short downstream intron (green) consistently results in a lower inclusion rate (Figure 3C, D and E respectively). Modulating *k_p_*_(*i*–*j*)_ (Figure 3C) tuned the inclusion from ∼72 to 96% for the model gene with a long downstream intron (blue and red), and 69–79% for the gene with a short downstream intron (green).

Furthermore, k_3′(*i*)_ is a more sensitive parameter than k_5′(*i*)_ (Figure 3D and E): for the model gene with a long downstream intron, modulating k_5′(*i*)_ tuned the inclusion from 78 to 94%, and modulating k_3′(*i*)_ tuned the inclusion from ∼76 to 98% (Figure 3D and E, blue and red). This difference was even stronger for the gene with a short downstream intron: modulating k_5′(*i*)_ tuned the inclusion from 75 to 83%, and modulating k_3′(*i*)_ tuned the inclusion from 74 to 92% (Figure 3D and E, green). These simulations suggest that in the context of CTS, exons with short downstream introns are particularly regulatable as potential cassette exons via control of the 3′ splice site.

**Figure 4.**
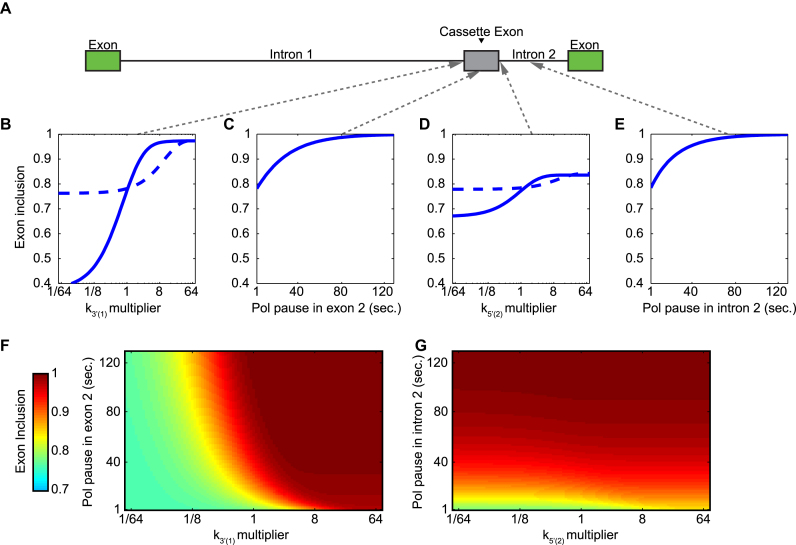
Dynamic range of exon inclusion displays differential sensitivity to 5′ and 3′ dynamics. (**A**) Schematic diagram of example gene used for simulations in lower panels. Cassette exon is indicated in gray and dashed arrows indicate locations of modulations performed in panels (**B**–**E**). (B) Cassette exon inclusion as a function of *k*_3′(1)_*. k*_3′(1)_ was varied throughout the entire simulation (solid line) or during a 1-s pause immediately after the 3′ splice site was synthesized (dashed line). (C) Cassette exon inclusion as a function of pause time in exon 2. (D) Cassette exon inclusion as a function of splice site availability (*k*_5′(2)_). *k*_5′(2)_ was varied throughout the entire simulation (solid line) or during a 1-s pause immediately after the 5′ splice site was synthesized (dashed line). (E) Cassette exon inclusion as a function of pause time in intron 2. (**F** and **G**) Cassette exon inclusion as a result of varying pausing and splicing parameters concurrently. (F) *k*_3′(1)_ of intron 1 was varied during a pause in the transcription of exon 2, whose duration was also varied. (G) *k*_5′(2)_ of intron 2 was varied during a pause in the transcription of intron 2, whose duration was also varied.

Since cassette exons are differentially included across tissue types or conditions, we hypothesized that there are kinetic conditions that render an exon more or less susceptible to regulation by splicing factors or other mechanisms. We aimed to find conditions where one parameter change could elicit a large dynamic range of inclusion rates in a model gene (Figure 4A), focusing on the splice sites at the junctions of the cassette exon. The rate of the internal 3′ splice site availability (k_3′(1)_) elicits a greater dynamic range of exon inclusion (37–97%: Figure 4B solid lines) than the rate of internal 5′ splice site availability (k_5′(2)_) or slowing the polymerase within exon 2 or intron 2 (67–83%, 78–100%, 78–100%, respectively) (Figure 4C–E, solid lines).

The model also allows us to explore the consequences of a slowed transcriptional elongation rate during which the availability rates of the splice site are altered (Figure 4B–E, dotted lines, Figure 4F and G). A pause immediately after synthesizing the 3′ splice site can act in concert with a temporary increase in the 3′ availability rate to vastly increase the inclusion rate of the exon (Figure 4F). In contrast, a pause immediately after the 5′ splice site of the second intron acts independently of the rate of 5′ availability (Figure 4G).

These model simulations suggest that inclusion or exclusion of an exon that have short downstream intron may be nimbly regulated by alterations of 3′ splice site accessibility of the upstream intron as well as polymerase pausing within the exon or downstream intron and that these mechanisms may function together.

### Intron and exon definition reinforce default splice patterns of distinct genes

**Figure 5.**
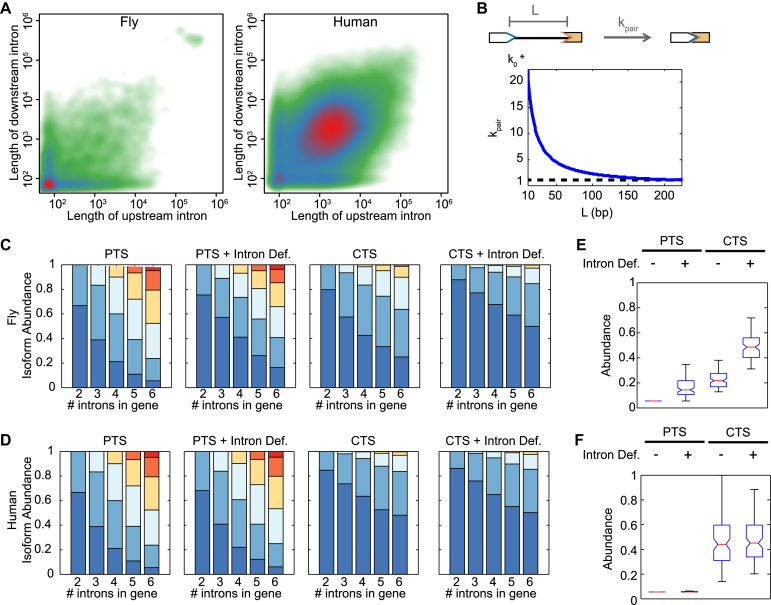
Intron definition is predicted to improve splicing fidelity additively with CTS in *Drosophila* genes, but not most human genes. (**A**) Density scatterplot of the lengths of the flanking introns of each internal exon in the *Drosophila* genome (left) and human genome (right). Green, blue and red indicate low, medium and high density, respectively. (**B**) Diagram (top) of intron definition in simulations plotted (bottom) for *k_p_* as a function of intron length (*L*). (**C** and **D**) Simulations of splicing fidelity of genes with 2–6 introns in *Drosophila* (C) and human (D) genomes. One hundred genes were selected from each category. Stacked bar plots of the average probabilities that the genes skipped 0, 1, 2, 3, 4 or 5 exons. All genes were simulated either with PTS (instantaneous elongation) only, with PTS and intron definition, with CTS, and with CTS and intron definition. (**E**) Distributions of the ‘perfect fidelity’ isoforms (0 exons skipped) in the 6-intron fly genes simulated in (C). (**F**) Distributions of the ‘perfect fidelity’ isoforms (0 exons skipped) in the 6-intron human genes simulated in (D).

Exons that are separated by a short intron are often quickly spliced together (31), supporting a model of ‘intron definition’ (25). Due to the size of fly introns, this phenomenon is more prevalent in fruit fly than in human transcripts (29) (Figure 5). We tested whether our model of AS is able to reproduce this phenomenon. To do so, we increased the joining rate across short introns (after their splice sites are available: Figure 5B). To test this interpretation we modeled genomic splicing patterns in fly and human genes using the CTASsimple model. First, we confirmed that CTS largely contributes to default splicing patterns (including all exons) compared to PTS (Figure 5C and E, first and third panels). Next, we tested the splice pattern of 6-intron genes with intron definition, and found that increasing the joining rate across short introns greatly improves fidelity (including all exons) in fly transcripts (Figure 5C and E, first versus second and third versus fourth panels), but much less in human transcripts (Figure 5D, F, first versus second and third versus fourth panels). Summary statistics are shown in panels (E) and (F) by plotting the abundance of fully spliced, ‘high fidelity’ transcripts that contain all exons: in the fly, intron definition improves the abundance of these high fidelity transcript in both PTS conditions, and even more so in CTS conditions (Figure 5D). However, in human, intron definition mechanisms make little difference, though CTS conditions markedly improves the fidelity of splicing (Figure 5E). In other words, this kinetic model-aided analysis supports the notion that intron definition is a more prevalent mechanism in the fly than in the human genome.

**Figure 6.**
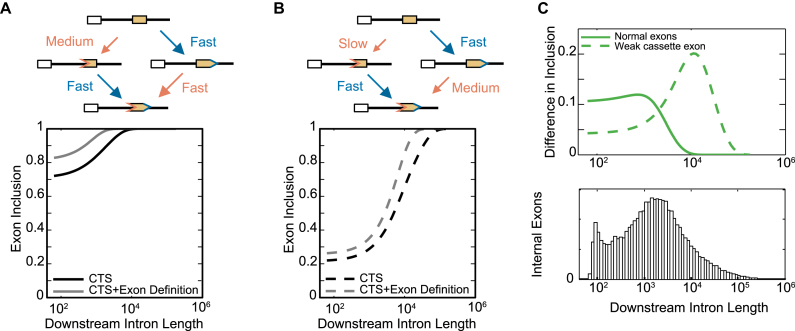
Model of exon definition accurately predicts optimal lengths of human introns. (**A**) Exon definition simulations defined for ‘normal’ exons, which are usually included in human transcripts: *k*_3′(1)_ is slower than *k*_5′(2)_, but *k*_3′(1)_ conditionally becomes fast when the 5′ site is already available (top). Bottom: Simulations of exon inclusion as a function of downstream intron length without the conditional speed up of *k*_3′(1)_ (‘CTS only’, blue), or with the conditional speed up (‘exon definition’, red). (**B**) Exon definition for weak cassette exons, where 3′ availability starts unusually slowly (with other aspects the same as in panel A). (**C**) Top: Difference in exon inclusion for normal (as in (A), solid green line) and weak (as in (B), dashed green line) internal exons as a function of downstream intron length. Bottom: histogram of intron lengths downstream of internal exons (i.e. non-first introns) in the human genome.

Exons flanked by two large introns dominate the human genome (Figure 5A). In general, exons are short, but when very long, they are included to a lesser degree, i.e. ‘exon definition’ (24). One hypothesis posits that when exons are short, interactions between splice sites across the exon may lead to a higher rate of inclusion (32). Our model of CTS may underestimate the precision of splicing in the human genome, and thus we examined to what degree exon definition might improve the inclusion of all exons. We implemented exon definition by formulating a 3′ internal splice site availability reaction that is stimulated by the availability of the 5′ splice site across the exons. We then tested the effect of this perturbation in model exons with a wide range of downstream intron lengths (Figure 6A), and in particular for weak cassette exons (Figure 6B). For both classes of exons, exon definition had a modest effect, suggesting that when downstream intron lengths are so short that they disfavor exon inclusion, exon definition increases exon inclusion. When plotting the gain in exon inclusion due to exon definition over the actual range of human introns, we found the largest effect for weak cassette exons that had very long downstream introns (Figure 6C, top panel). Interestingly, weak cassette exons are known to be flanked by long introns in the human genome (33). Indeed, in our analysis the distribution of intron lengths flanking weak cassette exons is similar (though shifted a little to the left) of what the model predicted would be affected to the greatest degree by exon definition (Figure 6C, bottom panel).

## DISCUSSION

The development of next generation sequencing methods has dramatically expanded the study of RNA processing to a genome-wide scale, where genomic, epigenomic and a variety of transcriptional or post-transcriptional mechanisms may be integrated. To develop an understanding of these nuclear events and interpret these genome-wide datasets, theoretical frameworks or quantitative models are needed that allow integration of diverse, high-dimensional information. Here, we constructed two alternative kinetic models of CTAS. The first, more detailed CTAS model includes reactions occurring at splice sites that are required as a prerequisite to two splice sites being properly paired. This formulation permits the modeling of complex phenomena such as intron and exon definition, as well as splicing factor recruitment by the elongating Pol II. However, the necessary combinatorial complexity of this model limits its use to studying splicing events that occur between no more than six splice sites (three introns). As a complement, we developed a reduced model (CTASsimple) with which we are able to extend simulations on genes of up to nine introns. Since the median number of introns per gene in the human genome is 7.8 (34), the CTASsimple model can be used to study quantitatively the mRNA isoform levels seen in the majority of human genes. Both models can be used to test hypotheses about how AS is controlled, as it occurs in the dynamic context of polymerase activity, i.e. as a co-transcriptional process, by considering specific segments of genes for modeling.

The results demonstrate that exon inclusion is favored when splicing is co-transcriptional rather than post-transcriptional (Figure 2), and that CTS alone could largely account for the relatively low overall exon skipping observed in the human genome (Figure 5E). In other words CTS but not PTS favors the generation of one particular default mature transcript in which all exons are included, whereas PTS will in principle result in all combinations of exons, including many that are likely non-functional. As a result, one may expect that in genomes or genes that are expressed primarily via PTS, splicing may rely on strong activating trans-factors in the context of poor basal splice-site activities to favor a specific functional transcript and minimize the fraction of non-functional transcripts. In contrast, in conditions where CTS is dominant, the default transcript does not require the involvement of regulatory splicing factors; but AS control will likely involve both activating and repressing splicing factors to favor the alternative transcript and repress the default transcript. While this remains theoretical conjecture, exploring it may provide insights about the principles of splicing control of specific gene(s), and may even shed light on the evolution of splicing as a co- or post-transcriptional molecular mechanism.

Within the kinetic modeling framework we were able to account for key aspects of the intron- and exon-definition hypotheses. These hypotheses are based on biochemical studies of cell free experimental systems, but they have not been quantitatively related to genome-wide *in vivo* data. Using the CTASsimple model, we selectively modified the pairing rates across introns and found thus that intron definition potentially contributes to achieving high splicing fidelity in the *Drosophila* genome, but not in the human genome, as previously observed (29). Conversely, using the full CTAS model, which encodes splice-site availability reactions, we predicted that most human exons will be included at 12% greater frequency than they would be without the benefit of exon definition, and that weak cassette exons surrounded by long introns would be included at up to 20% greater frequency. These effects are not large, but they are comparable to those achieved by RNA-binding proteins on endogenous genes (35). Remarkably, alternatively spliced human cassette exons tend to be flanked by long introns and have weak splice sites (33), and are predicted to be included by means of exon definition. Thus, the model constructed here was able to account for and predict the biologically relevant conditions for which exon definition has the most phenotypic importance.

Other genome-wide studies of the impact of RNA polymerase speed on splicing, using ‘fast’ and ‘slow’ RNA polymerase mutants, also support a model whereby polymerase speed influences co-transcriptional exon inclusion (36). Intriguingly, there are some exons whose inclusion is affected by both fast and slow polymerases, suggesting that these may be dependent on ‘optimal’ elongation rates. One can imagine that such rates might affect exon inclusion in difficult to predict ways, e.g. protein association with the pre-mRNA, protein association with other components of the spliceosome, formation of RNA secondary structure, etc. While the underlying mechanisms governing the inclusion of these exons may not be captured by the current model, iterative refinement of the model may help to understand the relative contributions of these parameters to exon inclusion.

The model described here also nicely accounts for splicing outcomes driven by exon definition for cassette exons and long introns. However, there have been intriguing reports from analysis of splicing in *Drosophila* (37,38) and mammals (39,40) of recursive intron excision, whereby an intron is removed in several steps before the two flanking exons are spliced together. Although the present model does not recapitulate iterative intron excision, it can be expanded to explore multi-step recursive splicing when the location of cryptic splice sites are known.

The majority of AS events in the human genome are simple exon skipping events (41). Our full CTAS model is well suited for quantitative studies of such splicing events in greater mechanistic detail than the CTASsimple model is capable of achieving. Using this model, we examined the relationship between the rates of 5′ and 3′ splice site availability and gene structure. In our example genes, a relatively short intron downstream from a cassette exon substantially increases the likelihood that the exon is skipped, and inclusion can be rescued by rapid 3′ splice site kinetics but not rapid 5′ splice site kinetics (Figure 3). Additionally, we found that in such genes, exon inclusion is quite sensitive to the kinetic rate of the internal exon's 3′ splice site availability (Figure 4B). Consistent with these model predictions, it has been reported that the spliceosomal U1 small nuclear ribonucleoprotein (snRNP) is recruited rapidly to the 5′ splice site (42), and U2 snRNP is recruited to pre-mRNA more slowly indicating that this step is rate-limiting. This is consistent with the adenosine triphosphate (ATP) requirement of this step.

The models developed here serve as a kinetic framework that can be extended to study the role of Pol II elongation dynamics, trans-acting splicing factors, or chromatin states; as well as other forms of AS such as the use of cryptic splice sites, on RNA processing outcomes. We have shown that mapping RNA Pol II dynamics to reaction rates or elongation times in our model, it is possible to simulate RNA Pol II-mediated modulation of splice site choice. For example, if RNA Pol II pauses immediately after the internal 3′ splice site and simultaneously increases its reaction rate, inclusion of the exon is dramatically increased (Figure 4F). Further, the complementary nature of the full CTAS and the CTASsimple models provide a framework for iterative refinement, taking observations from detailed kinetic analysis of individual splicing events or cassette exons obtained with the full model and feeding those revelations into CTASsimple model to create a more accurate view of splicing along an entire transcript in the context of detailed kinetic insight; with subsequent results informing inputs or specific splicing events to further examine and clarify with the full model.

This type of approach could be especially useful in addressing currently unresolved questions in the field, such as inferring mechanisms by which splicing factors achieve their effects. Most current experimental model systems examine the function of splicing factors in static gene models (i.e. equivalent to PTS), despite the fact that the majority of splicing is co-transcriptional or at least initiated during the transcription phase. The CTAS model framework presented here may aid in the interpretation of datasets obtained from cells with genetic perturbations of a splicing factor, and further mechanistic insight may be gleaned by model simulation of trans-factor events in a CTS framework rather than relying exclusively on statistical approaches. Indeed, the greatest advances in inferring splicing regulation may result from models that combine the co-transcriptional kinetics explored here with machine-learning and statistical approaches that incorporate *cis-* and *trans-*elements specific to each event (5,6).
