# Mathematical modeling identifies potential gene structure determinants of co-transcriptional control of alternative pre-mRNA splicing

**DOI:** 10.1093/nar/gkz013

**Published:** 2019-01-15

**Authors:** Jeremy Davis-Turak, Tracy L Johnson, Alexander Hoffmann

**Affiliations:** 1 San Diego Center for Systems Biology (SDCSB), University of California, San Diego, La Jolla, CA 92093, USA; 2 Department of Molecular, Cell, and Developmental Biology, University of California, Los Angeles, CA 90095, USA; 3 Molecular Biology Institute (MBI), University of California, Los Angeles, Los Angeles, CA 90095, USA; 4 Department of Microbiology, Immunology, and Molecular Genetics (MIMG), University of California, Los Angeles, CA 90095, USA; 5 Institute for Quantitative and Computational Biosciences (QCB) University of California, Los Angeles, Los Angeles, CA 90095, USA

*Nucleic Acids Research*, 2018, 46(20): 10598–10607. https://doi.org/10.1093/nar/gky870

The Authors apologize for several errors in the text. Corrections are highlighted below in bold:

## MATERIAL AND METHODS

### Model description

The model is represented as a Markov Chain (MC) with transition matrix Q, and is always a Directed Acyclic Graph (DAG). The root node represents the premature transcript with all introns present and all other nodes represent unique states of splicing outcomes. The absorbing states in the MC are all fully spliced products. The edges connecting the nodes represent splicing-related reactions, either splice site availability or pairing reactions. Availability reactions at 5′ and 3′ splice sites of introns a and b proceed with rates k_5′(a)_ and k_3′(b)_, respectively. Pairing reactions between a 5′ splice site of exon a and 3′ splice site of exon b (subject to b ≥ a) can proceed (at rate k_pa-b_) only if the availability reactions have occurred at the respective splice sites.

For a gene with N introns, there are N−1 internal exons, and thus **2^N−1^** possible fully spliced products, because each internal exon can either be included or excluded. Each of the **2^N−1^** fully spliced isoforms is represented by a subset S_f_ of absorbing nodes. All such subsets S_k_∀ k ∈ [1, 2, …, **2^N−1^**] are non-overlapping. This interpretation is necessary because for each of n exons that was skipped in a given isoform, there are 2 splice sites adjacent to that exon that were not used in any splicing reaction, yet an availability event may have occurred at each of those splice sites. Thus the subset S_k_ for isoform k that skips n exons contains 4n nodes.

Each node x is represented by a lexographically ordered series of reactions that it has undergone. Let R be an upper diagonal matrix entries of all the pairing reactions. R_ij_ represents a pairing of the 5′ splice site from intron i to the 3′ splice site of intron j: i ≤ j. A constitutive splicing reaction occurs when i = j. There are N(N + 1)/2 splicing reactions possible. We denote the identity of each node by a tuple of availability reactions states and entries into R. For example, a splicing intermediate in which intron 1 is constitutively spliced and the donor of intron 2 has spliced to the acceptor of intron 3, and the availability reactions have not occurred at the unused splice sites, is denoted

\begin{equation*}{{\boldsymbol{x}}_{{\boldsymbol{r}}_1^5,{\boldsymbol{r}}_1^3,{\boldsymbol{r}}_2^5,{\boldsymbol{r}}_2^{ - 3},{\boldsymbol{r}}_3^{ - 5},{\boldsymbol{r}}_3^3,\left( {1,1} \right)\left( {2,3} \right){\boldsymbol{\ }}}}\end{equation*}

where ${\boldsymbol{r}}_{\boldsymbol{j}}^5{\boldsymbol{\ }}$and ${\boldsymbol{r}}_{\boldsymbol{j}}^3$ indicate recruitment at the 5′ and 3′ splice sites, respectively, of intron *j* have occurred, and ${\boldsymbol{r}}_{\boldsymbol{j}}^{ - 5}{\boldsymbol{\ }}$ and ${\boldsymbol{r}}_{\boldsymbol{j}}^{ - 3}$ indicate that they have not.

The published article has been updated.

